# Empowering leadership during the COVID-19 outbreak: Implications for work satisfaction and effectiveness in organizational teams

**DOI:** 10.3389/fpsyg.2023.1095968

**Published:** 2023-03-17

**Authors:** Erik Eduard Cremers, Petru Lucian Curşeu

**Affiliations:** ^1^Department of Organization, Open Universiteit, Heerlen, Netherlands; ^2^Department of Psychology, Babeş-Bolyai University, Cluj-Napoca, Romania

**Keywords:** empowering leadership, team effectiveness, work satisfaction, leadership support, COVID-19, resilience

## Abstract

The COVID-19 pandemic generated unprecedented challenges for social and organizational life. We set out to explore how empowering leadership and leadership support were affected as a result of the team-based organization starting to implement flexible and remote work practices after the outbreak of the COVID-19 pandemic. We collected data in a cross-lagged design and used the two-condition MEMORE mediation procedure to analyze data on work satisfaction and team effectiveness obtained just before and immediately after the COVID-19 outbreak in 34 organizational teams. Our results show that the COVID-19 outbreak did not significantly impact perceptions of empowering leadership or perceived leadership support. However, teams that experienced changes in empowering leadership also reported proportional changes in work satisfaction and effectiveness. Finally, we show that the association between empowering leadership and leadership support, on the one hand, and work satisfaction in teams, on the other hand, is moderated by team size, such that the strength of the association is higher in small rather than large organizational teams. We conclude by arguing that the team-based organization absorbed well the impact and disruptions associated with the COVID-19 outbreak. We also stress the role of empowering leadership as a driver of work satisfaction and the effectiveness of organizational teams.

## 1. Introduction

As the COVID-19 outbreak spread across the world, many organizations had to implement flexible and remote work practices almost instantly and on a large scale. In this respect, the pandemic imposed increased pressure on employees working in organizations and teams, suddenly having to work at home full-time (Pluut and Wonders, 2020). This generated multiple challenges in terms of integrating family and work life in the home domain (Nikolova et al., 2021; Ratiu et al., 2022), yet also raised relational challenges for the way organizational teams planned and coordinated their actions (Contreras et al., 2020; Blanchard, 2021; Karl et al., 2022).

In a similar vein, leaders and managers were suddenly confronted with the challenge of leading from a distance in this new setting and attempting to fulfill leadership functions *via* online communication tools (Contreras et al., 2020; Chamakiotis et al., 2021; Coun et al., 2021). Both leaders and followers had to adapt to the new relational context, which created challenges for the quality of social exchanges and the provision of social support. On the one hand, followers needed more than ever support from their leaders and empowerment to adapt to the new working conditions, while, on the other hand, leaders often struggled to find effective ways to fulfill their roles.

We set out to explore how changes in the empowering leadership behaviors and leadership support triggered by the flexible and remote working during the COVID-19 outbreak affected work satisfaction and the effectiveness of organizational teams. Empowering leadership in teams involves delegating authority to and sharing power with the team members, stimulating participation in decision–making, offering support, and fostering autonomy in making decisions and performing tasks (Lee et al., 2018; Wang, 2022). Leadership support is a form of social support that includes instrumental (provision of advice and assistance in task accomplishment) and emotional (in terms of resolving conflicts at work or dealing with work strain) help that leaders offer to their followers (Contreras et al., 2020; Tummers and Bakker, 2021; Muntean et al., 2022). Research to date has not directly explored at the team level how changes in empowering leadership and leadership support due to the COVID-19 outbreak impact these team outcomes. We fill in this gap by using the results of a survey aimed at evaluating team dynamics in a large organization, to test the changes induced by the COVID-19 outbreak on perceived empowering leadership and supportive behaviors and indirectly on work satisfaction and effectiveness in teams. In this way, our article presents one of the first empirical attempts to directly test the influence of the COVID-19 outbreak and remote working prescriptions on the interplay between leadership behaviors, leadership support, and team outcomes.

## 2. Theoretical framework and hypotheses

With the COVID-19 outbreak, many organizations had to implement flexible and remote work practices almost instantly and on a large scale. This sudden change raised challenges not only for the organization as a whole but also for teams (Blanchard, 2021; Garro-Abarca et al., 2021) and individuals (Ratiu et al., 2022). At an individual level, beyond the ruminations related to the COVID-19 threat to personal wellbeing (Nikolova et al., 2021), employees quickly had to cope with combining work and private life in a full-time at-home setting, triggering pressures for employees and families (Pluut and Wonders, 2020). Work satisfaction refers to a set of evaluative cognitions related to the work environment that reflect a positive (cognitive) outlook toward the job and predominantly positive emotions experienced at work by employees (Horoub and Zargar, 2022). At the individual level, work satisfaction is one of the key indicators of wellbeing at work, as driven by empowering and participative leadership (Tummers and Bakker, 2021; Wang et al., 2022). Overall, teams were challenged to quickly adapt and find ways of effectively working together, now fully online. Team effectiveness is a multidimensional construct that captures the outcomes of team functioning in terms of productivity and viability (Mathieu et al., 2008), and these dimensions were certainly impacted by the change in work practices triggered by the COVID-19 pandemic (Edelmann et al., 2020; Blanchard, 2021; Garro-Abarca et al., 2021). Certainly, technology played a huge part in facilitating work and coping with the pandemic outbreak, but work practices, communication, and collaboration also had to be adapted to the new conditions (Blanchard, 2021). With such changes, team effectiveness and individual work satisfaction were also severely challenged and threatened. Considering the scale and immediacy of the global pandemic, we expect the COVID-19 crisis and its challenges for work settings to decrease work satisfaction and team effectiveness. Therefore, we hypothesize that,

*Hypothesis 1: The outbreak of the COVID-19 pandemic decreased work satisfaction in organizational teams*.
*Hypothesis 2: Remote working established during the outbreak of the COVID-19 pandemic decreased team effectiveness*.

Modern organizations increasingly rely on teams and multiteam systems to perform effectively and stimulate autonomous work (Meslec et al., 2023), and they tend to change their leadership approaches from hierarchical forms to more participative and empowering ones. Leadership support is a key antecedent for the effectiveness of organizational teams as it impacts job crafting and is a key job resource for employees (Cortellazzo et al., 2019; Fodor et al., 2021; Tummers and Bakker, 2021). In particular, empowering leadership and leadership support are job resources that ultimately translate into employee satisfaction and team effectiveness (Tummers and Bakker, 2021; Wang et al., 2022). Empowering leadership focuses on distributing power to employees and creating conditions for autonomous work with the goal of increasing motivation and effectiveness at work (Lee et al., 2018; Horoub and Zargar, 2022). Organizations and teams using empowering initiatives perform better than those relying on more traditional hierarchical structures, indicating an important need for empowering leadership in modern work settings, especially when using teams (Sharma and Kirkman, 2015). Meta-analytic evidence shows that empowering leadership is beneficial for work performance and creativity at the individual level as well as at the team level of analysis (Lee et al., 2018). Empowering leadership also influences employee attitudes and fosters job satisfaction (Sharma and Kirkman, 2015; Horoub and Zargar, 2022). Furthermore, empowering leadership has been identified as a dominant modern perspective in team leadership and one of the key antecedents of team effectiveness (van Knippenberg et al., 2021), as it drives team meaningfulness (Lisak et al., 2022) and constructive deviance (Wang, 2022). Furthermore, the meta-analytic study by Lee et al. (2018) showed that the beneficial effects of empowering leadership are channeled through psychological empowerment, trust in the leader, and the quality of leader-member interactions and exchanges.

As the COVID-19 outbreak also generated challenges for leaders and managers in modern workplaces and their empowering leadership (Lisak et al., 2022; Wang, 2022; Wang et al., 2022), we expect that changes in empowering leadership practices during COVID-19 indirectly impacted team effectiveness and work satisfaction. A recent study that explored the relationship between empowering leadership and innovative work behaviors of employees after the COVID-19 outbreak showed that work-related flow mediated the association between empowering leadership and innovative work behavior (Coun et al., 2021). However, this study did not directly evaluate changes in empowering leadership practices triggered by the COVID-19 outbreak. As we stated before, we expect that empowering leadership practices will become less effective as interactions become mediated by virtual communication. Furthermore, we believe that the flexible and online work generated challenges in providing much-needed leadership support and ultimately reduced the satisfaction and effectiveness of organizational teams. To summarize, we expect that the possible detrimental effects of online work on work satisfaction and effectiveness in teams can be explained by the reduced quality of interactions with the leaders, diminishing leadership support, and the likely effectiveness of empowering leadership practices.

*Hypothesis 3: Change in empowering leadership and leadership support during the COVID-19 outbreak explain decreased work satisfaction in organizational teams*.
*Hypothesis 4: Change in empowering leadership and leadership support during the COVID-19 outbreak explain decreased team effectiveness*.

The shift from face-to-face meetings, including spontaneous unplanned ones to online pre-planned meetings during the outbreak of the COVID-19 pandemic generated important challenges for the development of team processes, mutual social support, cohesion, and entitativity of organizational teams (Blanchard, 2021). During the pandemic, team members lost the benefits of the spontaneous meetings and the social support they received at work, with a possible pervasive impact on work satisfaction in teams. Furthermore, the entitativity (the shared perception that the team is a unitary social entity) of teams was negatively affected by the transition to online interactions, with potential detrimental effects on work satisfaction (Blanchard, 2021). Given these arguments, we expect that smaller teams are more effective at rallying their relational resources to cope better with the diminishing social support and the dilution of entitativity generated by the transition to online working, while larger teams may experience difficulties in preserving their relational synergy. We have argued that COVID-19 and the transition to online working imposed constraints on empowering teams and we expect that the benefits of empowering leadership for work satisfaction in teams are preserved in small rather than in large teams. We expect that small teams will be more effective in coping with such a decrease in empowering leadership, as in small teams, members are more likely to effectively provide social support and maintain wellbeing at work in the team as compared to members in large teams.

Previous research already showed that team size influences the relationship between group performance and team leadership. One of the theoretical explanations for the differential effects of leadership depending on team size is the Social Impact Theory (Latané, 1981). Latané's psychosocial principle of “division of impact” predicts the dilution of leaders' social influence with the number of targets, that is, the members of the team. Furthermore, O'Connell et al. (2002) put forward a “contextualist” perspective of leadership, according to which leadership effectiveness is influenced by contextual factors such as team design and size. To summarize, these two explanations rely on the division and diffusion of leadership impact as the group size increases. In their discussion of differences between larger and smaller teams on technical tasks and creative tasks, Karriker et al. (2017) argued that the logistics of small teams are easier to manage. They also add that the relationship between smaller teams and improved goal attainment can be explained in light of the higher frequency of communication that occurs in these smaller teams. This increased communication frequency can reduce group conflict and support higher levels of shared understanding of the end goal (Karriker et al., 2017).

Smaller teams also develop more effective coordination processes, as it is easier for a few rather than many members to effectively synchronize their actions in a synergetic manner (Curşeu et al., 2017). Furthermore, the transition to online meetings generated important constraints in planning and organizing team meetings (Blanchard, 2021), yet such challenges are expected to be less impactful in smaller teams than in larger teams. We argued that change in empowering leadership triggers proportional changes in team effectiveness, and in line with the above arguments, we argue that team size moderates this association such that smaller teams are better able to cope with the process losses associated with the transition to online meetings after the COVID-19 outbreak. Therefore, we hypothesize that,

*Hypothesis 5: Team size attenuates the association between change in empowering leadership and leadership support due to the COVID-19 outbreak on the one hand and change in work satisfaction in organizational teams on the other hand*.
*Hypothesis 6: Team size attenuates the association between change in empowering leadership and leadership support due to the COVID-19 outbreak on the one hand and change in team effectiveness on the other hand*.

## 3. Methods

### 3.1. Sample and procedure

Our study is based on a cross-lagged design and was conducted in a large organization that initiated a team-based reorganization of work and the implementation of multiteam systems in 2019, with the initial aim to survey the changes triggered by this reorganization. The first wave of data collection among 34 organizational teams took place in January 2020 in a face-to-face, on-premise work setting. The second wave of data collection took place after the COVID-19 outbreak in June 2020 in a fully online work setting. This study is based on the differences observed for these 34 teams during these two data collection moments.

Participants were asked to fill in a survey evaluating different aspects of teamwork and work satisfaction, while team leaders were asked to evaluate team effectiveness on three main dimensions, namely, performance, innovation, and ownership. A total of 177 members (32 women) with an average age of 45.87 years filled in the survey during the first wave, and 125 participants (22 women) with an average age of 48.22 years filled in the survey during the second wave. Participation was voluntary and anonymous, and participants could withdraw from the study at any moment if they wished so. As the study documented the implications of team-based reorganization, all employees who were organized in teams across the different value streams in scope were invited to fill in the survey. Data on work satisfaction, empowering leadership, and leadership support were collected from the team members, while data on team effectiveness was collected from the team leaders (consisting of several value stream and team orchestrating roles that could independently evaluate team effectiveness). The scores obtained from team members were aggregated to obtain a team-level score, which we have used for further analyses of the teams for which we had evaluations at both time points. Team size was extracted from the company records. In short, we collected data from multiple sources for the variables included in the model, and all analyses were conducted at the group level.

### 3.2. Measures

*Work satisfaction* was evaluated by asking team members to answer two items: “All things considered, how satisfied are you with your work in general” (1–7) and “How happy are you feeling in your job in general” (0–10). Cronbach's alpha for this scale was 0.77 at time 1 and 0.78 at time 2. Given the fact that the items were evaluated using different Likert scales, we have used the dominant Bartlett factor score as an accurate indicator of the underlying work satisfaction component rated with the two items (DiStefano et al., 2009).

*Team effectiveness* was evaluated by asking team leaders to rate from 1 to 5 the *performance, innovativeness*, and *ownership* of each participating team. As the three items refer to different facets of team effectiveness, we have computed the omega reliability index based on factor analysis (Hayes and Coutts, 2020). At time 1, omega was 0.49, with the innovation item loading the least in the dominant factor score, while at time 2, omega was 0.72, with all items loading positively in the dominant factor score. For further analyses, we will use the Bartlett dominant factor score as an accurate index of team effectiveness, considered an underlying factor evaluated by the three ratings provided by the team leaders (DiStefano et al., 2009).

*Empowering leadership* was evaluated by team members with a single behaviorally anchored rating item, asking the participants to rate the leadership style of their leaders in and around the team on a continuum ranging from very restrictive (1 = provides specific guidelines that limit your choice of action) to empowering leadership (7 = gives autonomy and space to decide on how to perform your work). Therefore, a high score reflects empowering leadership.

*Leadership support* was evaluated with two items that capture instrumental and emotional support, presented in the study by Muntean et al. (2022), by asking team members to answer the extent to which they receive task-related and relational support from their leaders. The two items were “When I encounter problems around my tasks at work, I get the most help and directions from my manager(s)” and “When I experience relational problems at work, I get the most support from my manager(s)” (answers were recorded on a five-point Likert scale, from 1 = never to 5 = a lot). Cronbach's alpha for this scale was 0.68 for time 1 and 0.78 for time 2. We have further used the Bartlett dominant factor score for the analyses as an accurate indicator of the underlying factor evaluated by these two items (DiStefano et al., 2009).

*Team size* was evaluated by collecting data from the company records on the total number of members in each organizational team.

## 4. Results

**Figure 1:**
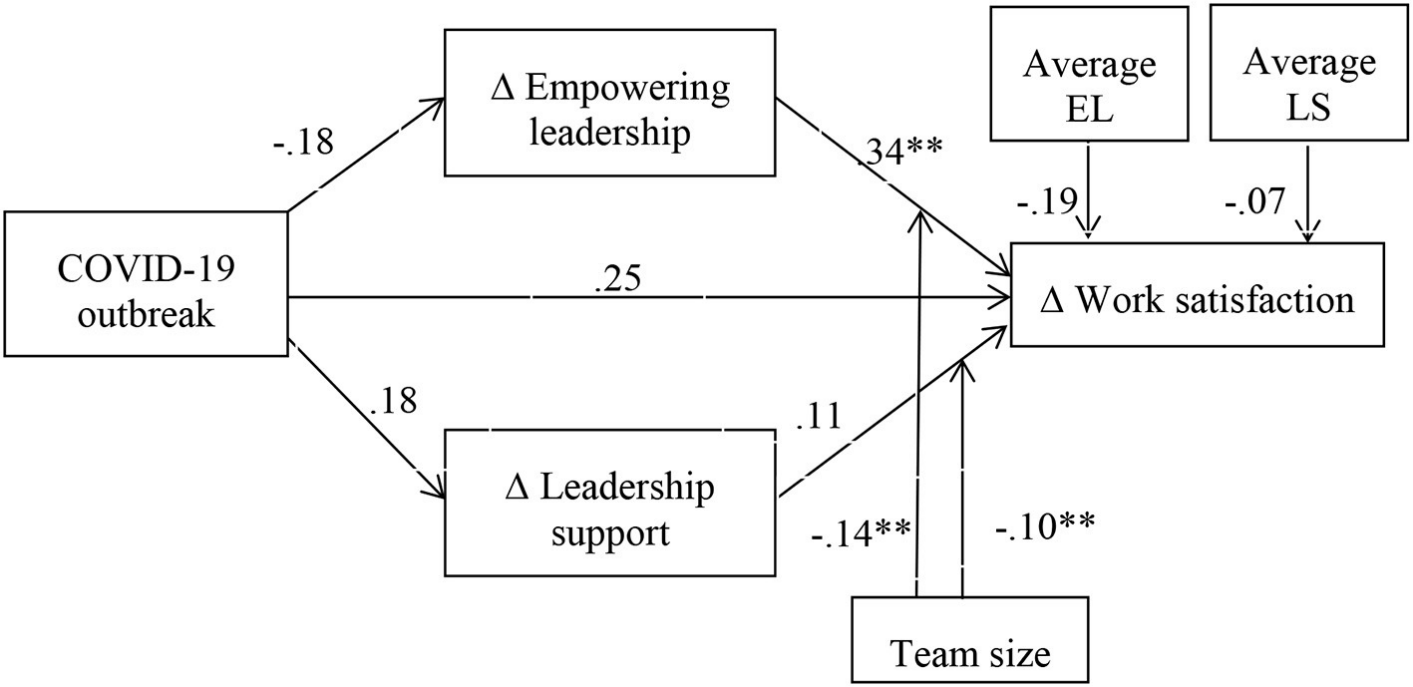
The overall moderated mediation results for change in work satisfaction as a function of change in empowering leadership and leadership support after the COVID-19 outbreak. **p < 0.01; EL, empowering leadership; LS, leadership support; Δempowering leadership, empowering leadership before COVID-19 minus empowering leadership after the COVID-19 outbreak (change in empowering leadership due to COVID-19); Δleadership support, leadership support before the COVID-19 outbreak minus leadership support after the COVID-19 outbreak (change in leadership support due to COVID-19); Δwork satisfaction, work satisfaction before the COVID-19 outbreak minus work satisfaction after the COVID-19 outbreak (change in wellbeing due to COVID-19).

Our design included two-time measurements, one just before the COVID-19 outbreak and one just after the outbreak, and the introduction of work at home in the organization. Given that we have this major event that occurred between the two waves of data collection, we will use the procedure to test mediation in designs with repeated measures as described in Montoya and Hayes (2017). This procedure allows the test of complex mediation and moderated mediation models in which the change in the dependent variable associated with an external event (the COVID-19 outbreak in our case) can be explained by the change in a mediating variable triggered by or associated with the same event (Montoya and Hayes, 2017; Montoya, 2019). Therefore, we modeled empowering leadership and leadership support as mediators and estimated the extent to which these scores changed at the team level after the COVID-19 outbreak. We also modeled work satisfaction and team effectiveness as dependent variables by estimating the change in these variables triggered by the same external event. We then used the MEMORE 3.0 macro for SPSS version 28 (Montoya, 2022) to analyze the data, using Model 16 that includes the moderating role of team size. The results of the overall moderated mediation analyses are presented in Figure 1 for work satisfaction and **Figure 4** for team effectiveness.

The results of the repeated measures mediation analysis reveal that the COVID-19 outbreak did not significantly reduce work satisfaction (effect size = 0.25; SE = 0.16, CI_low_ = −0.07; CI_high_ = 0.58) or team effectiveness (effect size = 0.26; SE = 0.16, CI_low_ = −0.05; CI_high_ = 0.58); therefore, hypotheses 1 and 2 were not supported by the data. Furthermore, the results also reveal that the COVID-19 outbreak did not significantly decrease teams' perceptions of empowering leadership (effect size = −0.18; SE = 0.20, CI_low_ = −0.60; CI_high_ = 0.24) or leadership support (effect size = 0.18; SE = 0.16, CI_low_ = −0.15; CI_high_ = 0.50). We can therefore conclude that the detrimental effects expected due to the COVID-19 outbreak did not emerge as hypothesized.

Furthermore, our results revealed a positive and significant effect of change in empowering leadership on change in work satisfaction (effect size = 0.34; SE = 0.11, CI_low_ = 0.10; CI_high_ = 0.56). This direct effect reveals that when empowering leadership increases, so does the work satisfaction reported in the teams. In other words, even though the COVID-19 outbreak did not have a systematic effect on change in empowering leadership, the teams that experienced such a change (due to other factors and circumstances) also reported a proportional change in work satisfaction.

**Figure 2:**
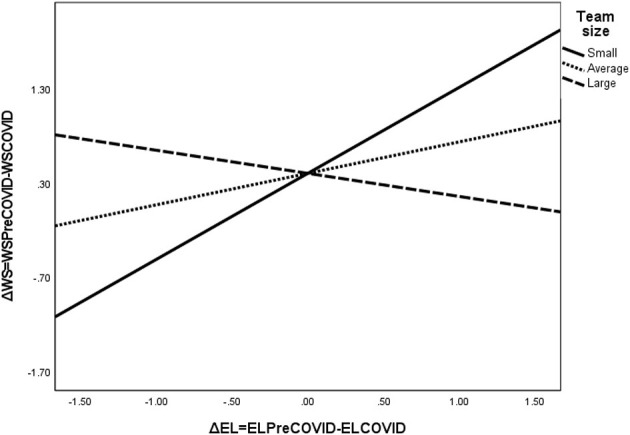
The interaction effect between changes in empowering leadership and team size on work satisfaction. ΔEL, change in empowering leadership due to COVID-19; ΔWS, change in work satisfaction due to COVID-19; ELPreCOVID, empowering leadership before the COVID-19 pandemic; ELCOVID, empowering leadership during the initial stages of the COVID-19 pandemic; WSPreCOVID, work satisfaction before the COVID-19 pandemic; WSCOVID, work satisfaction during the initial stages of the COVID-19 pandemic.

Furthermore, our results reveal significant interaction effects between change in empowering leadership and team size (effect size = −0.14; SE = 0.05, CI_low_ = −0.24; CI_high_ = −0.04), as well as between leadership support and team size (effect size = −0.10; SE = 0.03, CI_low_ = −0.16; CI_high_ = −0.04). The significant interaction effect between change in empowering leadership and team size reveals that the effect of empowering leadership change is positive and significant for small (effect size = 0.91, SE = 0.23, *p* = 0.0004, CI_low_ = 0.45; CI_high_ = 1.38) and medium-sized teams (effect size = 0.33, SE = 0.11, *p* = 0.0005, CI_low_ = 0.11; CI_high_ = 0.56), while the effect is negative but not significant for larger teams (effect size = −0.24, SE = 0.22, *p* = 0.27, CI_low_ = −0.69; CI_high_ = 0.20; see also Figure 2 for the illustration of the slopes).

**Figure 3:**
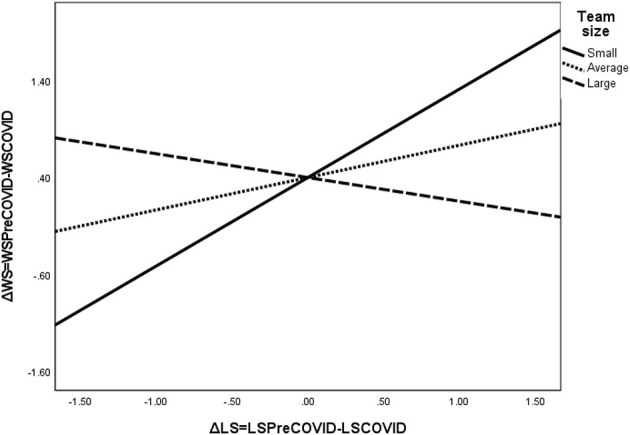
The interaction effect between change in leadership support and the team size on work satisfaction. ΔLS, change in leadership support due to COVID-19; ΔWS, change in work satisfaction due to COVID-19; LSPreCOVID, leadership support before the COVID-19 pandemic; LSCOVID, leadership support during the initial stages of the COVID-19 pandemic; WSPreCOVID, work satisfaction before the COVID-19 pandemic; WSCOVID, work satisfaction during the initial stages of the COVID-19 pandemic.

The interaction effect between leadership support and team size reveals that the association between change in leadership support and work satisfaction is positive and significant for small-sized teams (effect size = 0.53, SE = 0.20, *p* = 0.01, CI_low_ = 0.12; CI_high_ = 0.93), it is positive but not significant for average-sized teams (effect size = 0.11, SE = 0.14, *p* = 0.47, CI_low_ = −0.19; CI_high_ = 0.40), and it is negative and marginally significant for large teams (effect size = −0.32, SE = 0.18, *p* = 0.09, CI_low_ = −0.69; CI_high_ 0.06; see also the slopes depicted in Figure 3). Given these patterns of results, we can conclude that the moderating role of team size in the relationship between change in empowering leadership and leadership support, on the one hand, and work satisfaction in teams, on the other hand, was supported by the data, lending full support for hypothesis 5.

**Figure 4:**
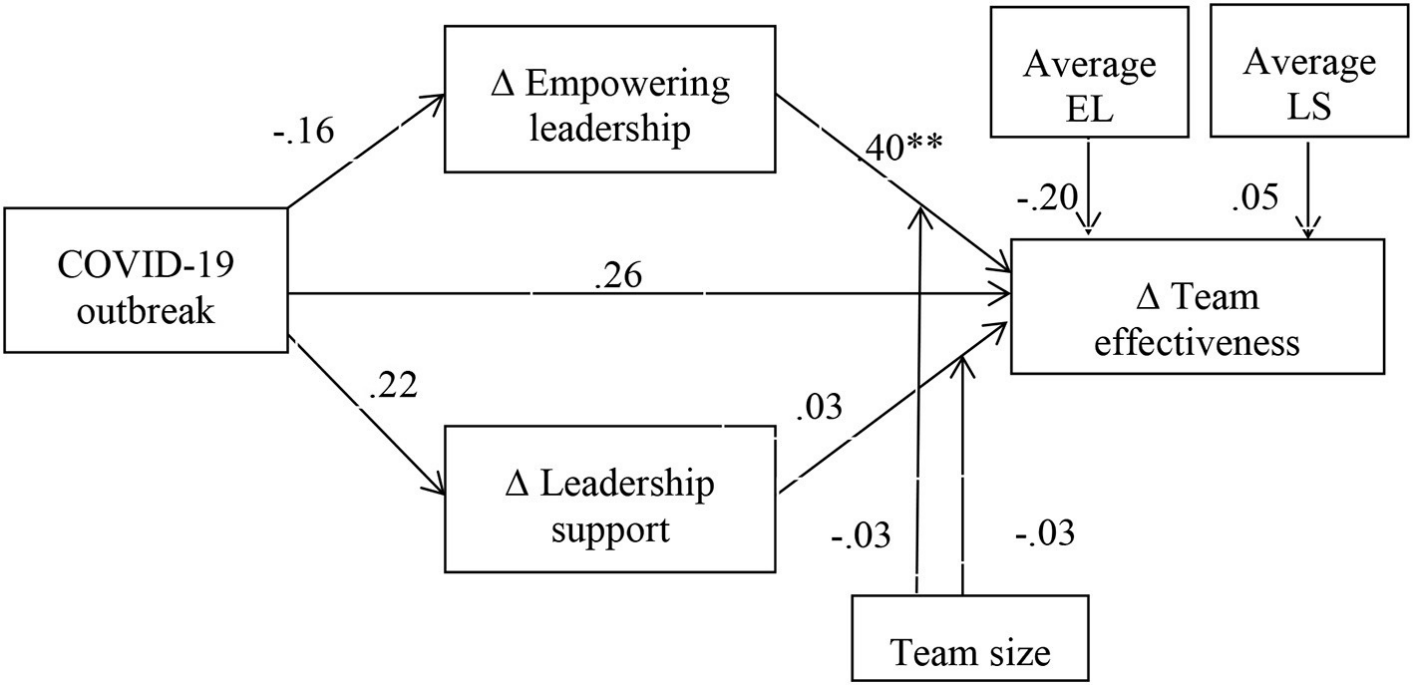
The overall moderated mediation results for change in team effectiveness as a function of change in empowering leadership and leadership support after the COVID-19 outbreak. **p < 0.01; EL, empowering leadership; LS, leadership support; Δempowering leadership, empowering leadership before COVID-19 minus empowering leadership after the COVID-19 outbreak (change in empowering leadership due to COVID-19); Δleadership support, leadership support before the COVID-19 outbreak minus leadership support after the COVID-19 outbreak (change in leadership support due to COVID-19); Δteam effectiveness, team effectiveness before the COVID-19 outbreak minus team effectiveness after the COVID-19 outbreak (change in team effectiveness due to COVID-19).

The moderated mediation results for team effectiveness as a dependent variable reveal a similar pattern of results, with the COVID-19 outbreak having no significant effect on any of the changes in the variables included in the model. The only significant effect is the positive association between the change in empowering leadership and the change in team effectiveness (effect size = 0.40, SE = 0.13, *p* = 0.006, CI_low_ = 0.12; CI_high_ = 0.68), and none of the interaction effects are significant. The overall results of this moderated mediation analysis with repeated measures are presented in Figure 4.

Given the fact that the outbreak of the COVID-19 pandemic did not have the expected detrimental effects (none of its main effects were significant), the mediating effects hypothesized in the third and fourth hypotheses were not supported by the data. Furthermore, as the moderating effects of team size in the relationship between change in empowering leadership and leadership support, on the one hand, and team effectiveness, on the other hand, were not supported (see Figure 4), we can conclude that hypothesis 6 was not supported by the data.

## 5. Discussion

This study aimed to capture the potential deleterious consequences of the COVID-19 outbreak on work satisfaction and team effectiveness as triggered by diminished empowering leadership and leadership support. We have used data collected before the COVID-19 outbreak and right after the outbreak in an organization that started using multiteam systems as a way of organizing teams. The analysis and results showed three relevant insights.

First, contrary to our expectations, the results of our study showed that in this organization, for the teams in scope, the COVID-19 outbreak did not significantly decrease work satisfaction or team effectiveness. Our understanding is that, in this regard, this organization and its teams seemed to have absorbed the shock of the COVID-19 outbreak quite well. The organization implemented an organizational setup based on delegating responsibilities to self-managing teams combined with more empowering leadership approaches to foster ownership and autonomous work. With teams established as a core and versatile unit for organizing work, this organization proceeded by introducing organizational constructs to aggregate multiple teams. The main reason for this organizational design was the pursuit of agility (Bundtzen and Hinrichs, 2021) and optimal use of human resources within the organization (Meslec et al., 2023). Based on the results of our study, we infer that this setup aimed toward agility also brought resilience. Resilience was illustrated by the capacity to handle a very disruptive crisis like the COVID-19 outbreak. Agility and resilience can be seen as two sides of the same coin, both addressing the challenges of increased VUCA (volatility, uncertainty, complexity, and ambiguity) environments (Bundtzen and Hinrichs, 2021), with agility in a more positive context and resilience in a more negative context. We did not directly evaluate team resilience, but we believe that the lack of disruptive effects in relation to this global threat is indirectly illustrative of resilience.

The results further show that the COVID-19 outbreak did not significantly decrease teams' perceptions of empowering leadership. In a similar study on changes in leadership behaviors associated with the COVID-19 outbreak, Stoker et al. (2022) showed that although leaders perceive they delegate more and control less, the employee perceptions are not necessarily aligned with what the leaders report. Much like our results, their study reports that employees do not perceive significant changes in the delegating behaviors of their leaders, but they do perceive a significant decrease in control (Stoker et al., 2022). However, the results of our study show the benefits of empowering leadership for work satisfaction and team effectiveness. These results are fully aligned with the ones reported by Coun et al. (2021), showing that in various stages of the pandemic, empowering leadership practices remained essential for innovative work. Similar results were reported by Siswanti and Muafi (2020), but these studies did not directly test the change in empowering practices triggered by the COVID-19 outbreak. This study addresses the need for more research on empowering leadership and leadership support during organizational and environmental changes (Horoub and Zargar, 2022; Wang et al., 2022) and uses two waves of data collected at the onset of an organizational change and separated by the COVID-19 outbreak to test directly the effects of empowering leadership and leadership support on team outcomes. Although our results show that empowering leadership did not change systematically as a result of the COVID-19 outbreak, when empowering leadership increases, so does the work satisfaction reported in teams. The same effect was found in the positive association between the change in empowering leadership and the change in team effectiveness. It is our understanding that in this organizational setup of teams and multiteam systems, empowering leadership is a positive and influential factor that fosters work satisfaction and drives team effectiveness.

A third important insight provided by the results of our study is the moderating role of team size on the influence of empowering leadership and leadership support on work satisfaction. The results show that the effects of empowering leadership change and leadership support change are positive and significant for small and medium-sized teams, while the effects are negative but not significant for larger teams. These findings are in line with the contextualist view on leadership (O'Connell et al., 2002) and Social Impact Theory (Latané, 1981). According to the Social Impact Theory (Latané, 1981), the effectiveness of social influence attempts decreases with the number of targets; therefore, we believe that such a “division of impact” explains the decreasing association between empowering leadership and leadership support with satisfaction in larger teams. In other words, the strength of the association between leadership support and empowering leadership, on the one hand, and work satisfaction, on the other hand, decreases with the number of team members in the team. In line with the contextualist view on leadership (O'Connell et al., 2002), the impact of empowering leadership and leadership support on satisfaction could also be diffused within the larger teams. Team size is a key design feature constraining the impact of leadership, with a nuanced insight from the results of our study that this applies to work satisfaction in teams but not to their effectiveness.

The three insights generated by the results, as discussed above, have implications for organizations looking for new ways of organizing and changing leadership practices. First, we show that using team-based organizational structures can play an important role in absorbing the effects of a major crisis like COVID-19 with regard to work satisfaction and team effectiveness. This organizational setup with teams as a core building block, aggregated and connected in a setup of multiteam systems, also generates the capability to absorb the impact of an environmental threat and thereby enhances organizational resilience. Second, an empowering leadership style is beneficial in team-based setups for fostering work satisfaction and driving team effectiveness. Finally, our results have implications for team design, as our results show that the beneficial role of empowering leadership for work satisfaction decreases with team size; therefore, managers should devote more attention to large teams in terms of leadership support and empowerment.

### 5.1. Limitations and future research directions

Our study has a few important limitations. First, the study relied on two data collection moments, and no manipulation for empowering leadership or leadership support was performed; therefore, we cannot draw definite causal conclusions concerning the associations reported in the article. We tested mediation using a repeated measures design, and as a result, we did capture the extent to which changes in empowering leadership and leadership support impact changes in team effectiveness and work satisfaction, but we cannot make definite causal claims about these associations. We join the voices calling for experimental research to disentangle the implications of empowering leadership for team dynamics and outcomes (Tummers and Bakker, 2021; Wang, 2022; Wang et al., 2022). Future studies could manipulate empowering leadership as well as leadership support and place teams in different experimental conditions to explore more directly the effects of these variables on team dynamics and outcomes.

Second, our study was conducted in a single organization that started implementing team-based structures, and as such, our results cannot be generalized to broader organizational settings. It is not unreasonable to assume that empowering leadership may not always be beneficial for team outcomes, especially in teams operating under uncertain or critical conditions in which role ambiguity is high. Empowering leadership fosters autonomy, yet in situations in which role ambiguity is high, empowering leadership could further accentuate role ambiguity and decrease performance (Sharma and Kirkman, 2015; Cheong et al., 2019). Future studies could try to explore the joint influences of empowering leadership and leadership support on team dynamics and outcomes in different teams that operate in volatile environments and have to perform complex tasks with fast-changing demands (such as military or crisis intervention teams). Furthermore, empowering leadership also has dark sides as it can push employees to excessively engage in pro-organizational behavior (Dennerlein and Kirkman, 2022). As pro-organizational behavior in excess has detrimental effects on performance and wellbeing because it increases workload as a job demand (Muntean et al., 2022), future studies could explore the dark sides of empowering leadership in team contexts.

Finally, we have evaluated empowering leadership using a single-item measure, and the reliability of such measures is rather limited. Future studies could use more elaborate and well-established measures of empowering leadership. More comprehensive measures of empowering leadership evaluated different facets (such as coaching, showing concern, leading by example, and participative decision-making; see Arnold et al., 2000), and these dimensions may have differential influences on team dynamics and outcomes. As most of the empirical studies to date have explored empowering leadership as a unidimensional construct (Cheong et al., 2019), future studies could explore the multidimensional influences of empowering leadership in teams.

## 6. Conclusions

Our study used a cross-lagged design aimed at surveying the implementation of teamwork in a large organization to test a mediation model in which changes in empowering leadership and leadership support triggered by the COVID-19 outbreak explain changes in team effectiveness and satisfaction. The two waves of our survey were separated by the COVID-19 outbreak, such that the first wave of data was collected just before the outbreak and the second wave of data was collected just after the COVID-19 outbreak. We were thus able to test, using mediation for repeated measures, the extent to which the mediation claims were supported.

The results provided three main insights. First, the COVID-19 outbreak did not significantly decrease work satisfaction or team effectiveness in this organization. Second, the COVID-19 outbreak did not significantly decrease teams' perceptions of empowering leadership or leadership support. However, results show empowering leadership as a positive and relevant factor in fostering work satisfaction and driving team effectiveness. A third insight concerns team size as a relevant team design feature constraining the impact of empowering leadership on work satisfaction.

Overall, we believe that using teams in an organizational setup absorbed well the disruptive impact of the COVID-19 pandemic on the satisfaction and effectiveness of organizational teams. Furthermore, empowering leadership is an important factor in driving work satisfaction and team effectiveness, especially in small- and medium-sized teams, while in large teams, its benefits are lower due to the division and diffusion of leadership impact.

## Data availability statement

The dataset analyzed for this study can be obtained from the corresponding author upon reasonable and motivated request.

## Ethics statement

The organizational representatives approved the study and the design of the study as well as the survey was reviewed and approved by the Ethical Review Committee of the Open Universiteit (Heerlen, the Netherlands). Surveys were translated in Dutch and participants gave their informed consent for participating in the study.

## Author contributions

EEC and PLC: study design, data analysis, first draft, and revisions. EEC: data collection. All authors contributed to the article and approved the submitted version.
